# Imaging of 3 bright terrestrial gamma-ray flashes by the atmosphere-space interactions monitor and their parent thunderstorms

**DOI:** 10.1038/s41598-024-57229-1

**Published:** 2024-03-23

**Authors:** Oscar A. van der Velde, Javier Navarro-González, Ferran Fabró, Víctor Reglero, Paul Connell, Olivier Chanrion, Jesús A. López, Joan Montanyà, Torsten Neubert, Nikolai Østgaard

**Affiliations:** 1 Electrical Engineering Department, Universitat Politècnica de Catalunya - BarcelonaTech, Terrassa, Spain; 2 Image Processing Laboratory, University of Valencia, Valencia, Spain; 3 Meteorological Service of Catalonia, Barcelona, Spain; 4 Space and Earth Science and Technology, Technical University of Denmark, Kongens Lyngby, Denmark; 5 Department of Physics and Technology, University of Bergen, Bergen, Norway

**Keywords:** Astronomy and planetary science, Particle astrophysics, Atmospheric science

## Abstract

The Atmosphere-Space Interactions Monitor (ASIM) on the International Space Station (ISS) includes an instrument designed to geolocate Terrestrial Gamma-ray Flashes (TGF) produced by thunderstorms. It does so with a coded aperture system shadowing the pixelated Low Energy Detector of the Modular X- and Gamma-ray Sensor (MXGS). Additionally, it locates associated lightning flashes with the Modular Multispectral Imaging Array (MMIA). Here we present 3 bright TGFs with very similar duration, fluency and observation distance. The innovative imaging capabilities allow us to determine the TGF position and correlate the TGF-lightning parent event in time and position simultaneously. The accurate position determination and distance to the observer allow us to perform a comparative study of TGF characteristics. These TGFs were produced in association with lightning flashes below the highest cloud tops of developing to mature convective cells. In one event, GLM (Geostationary Lightning Mapper) cloud flash rates were slowing down after the TGF while negative cloud-to-ground flashes suddenly ceased from 10 to 5 min before the TGF. An 8-stroke (strongest: -93 kA) cloud-to-ground flash occurred 31 s before the TGF. Vertical profiles from the ERA5 reanalysis data suggest TGFs may be produced in variety of tropical environments.

## Introduction

Terrestrial Gamma-ray Flashes (TGFs) were first discovered in 1991 by the Burst and Transient Source Experiment (BATSE)^1^, and have since been observed by various other instruments in space^2–6^. The various measurements performed by these instruments, not specifically designed for TGF detection, revealed that TGFs are the most intense radiation naturally produced on Earth with energies up to 40 MeV^3^. TGF durations vary from tens of microseconds to a few hundreds of microseconds^7,8^.

TGFs are produced within thunderstorms at altitudes between 9 and 15 km^9–11^ and they have been directly correlated with negative upward leaders^12–14^. Gamma rays in the TGFs are produced through bremsstrahlung radiation that results from the interaction of runaway electrons with air molecules. The required acceleration of electrons has been proposed to occur in the very strong electric fields created in the tips of lightning leaders^15–20^ and in the large-scale ambient electric field of thunderstorms^10,17^.

Another outstanding issue is our understanding of conditions within thunderstorms that favor TGF production, and the role of the meteorological environment. Studies that focused on the analysis of specific thunderstorms producing TGFs showed that TGFs are associated with tall tropical thunderstorms^21^ with high values of liquid water content and CAPE^22^ as well as large concentrations of cloud ice, cloud water, precipitating ice and rain droplets. On the other hand, no specific convection characteristic was found for TGF production^23^. Some studies found that TGFs frequently occur within 5 min from the peak of the lightning activity^24^, while others^25,26^ found a higher probability for TGFs to occur during declining flash rates with more powerful flashes. It was also reported^27^ that during the minutes preceding TGFs, longer charging intervals occur between strokes, compared to the minutes after. Climatological TGF studies^28,29^ have suggested a role for the altitude and distance between layers of oppositely charged particles in the cloud.

The Atmosphere-Space Interactions Monitor^30^ onboard the International Space Station is the first instrument specifically designed to perform a continuous monitoring of transients in the Earth’s atmosphere with an energy ranging from optical wavelengths to hard gamma rays (few eV to > 30 MeV). Its Modular X- and Gamma-ray Sensor (MXGS)^31^ was designed for gamma ray imaging and spectral analysis of TGFs while the Modular Multi-Spectral Imaging Assembly (MMIA)^32^ was designed for image analysis and high-speed photometry of associated lightning discharges. The ability of MXGS to locate the gamma ray source combined with the detection of associated lightning discharges by MMIA makes ASIM a unique instrument to better understand the origin of TGFs^31^ as demonstrated soon after it became operational in 2018^33^.

Since June 2018, more than 1000 TGFs have been recorded. In this paper we present a detailed analysis of three bright TGF observed shortly after the beginning of ASIM operations. They are located over Central America, Africa and the Indian Ocean (Table 1). We first discuss the properties of the TGF as detected by the MXGS Low Energy Detector (LED, 20 keV to 350 keV) and High Energy Detector (HED, 300 keV to 30 MeV) instruments and the TGF location using gamma ray imaging and MMIA information. Subsequently, we discuss the evolution of the thunderstorm cells, lightning activity and the meteorological environment that produced these three TGFs.

**Table 1:** TGF identification parameters.

TGF ID yy-mm-dd_DOY_OBS	Trigger time hh:min:sec	Duration (µs)	ISS latitude degrees	ISS longitude degrees
2018-08-30_242_1244	00:41:55.819334	204	4.455	+ 25.596
2018-09-01_244_641	08:19:54.219968	190	16.760	− 92.489
2018-09-04_247_19544	19:34:57.178860	241	− 0.628	+ 63.766

DOY stands for Day-Of-Year. The OBS number is the internal ID useful for L0 data retrieval at the ASIM database.

## Results: part I: TGF characteristics and imaging

### TGF light curves

**Figure 1:**
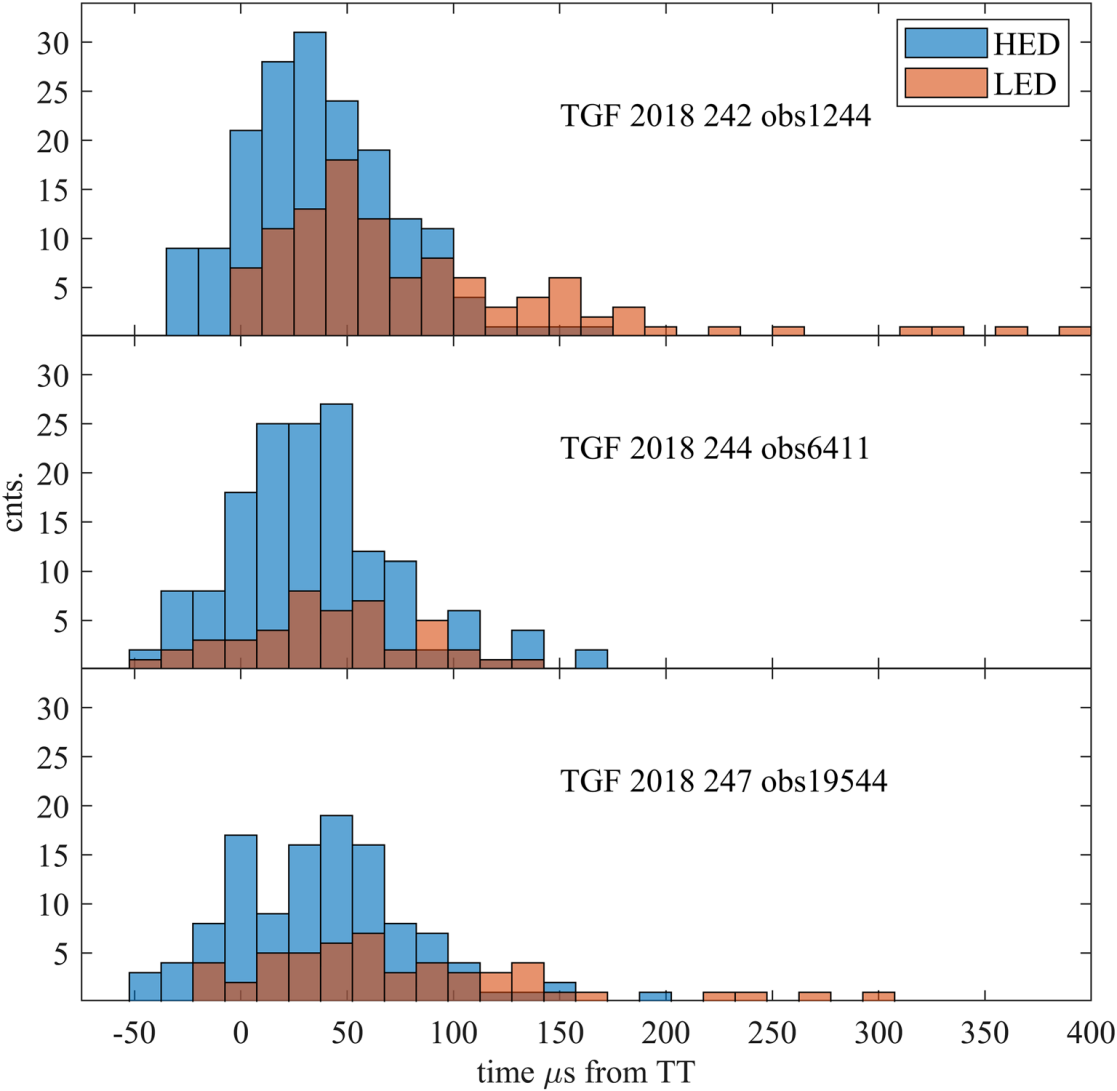
TGF light curves HED and LED for the DOY 242, 244 and 247 TGF. The origin of time is the TGF trigger time (TT).

TGF light curves for the three selected TGFs are displayed in Fig. 1. The origin of time is the TGF trigger time (TT). Table 2 summarizes the light curve main parameters.

**Table 2:** TGF light curve parameters.

DOY	Detector	Start µs	End µs	Duration µs	Max µs	Rise µs	Decay µs	Counts
242	HED	− 27	+ 177	204	+ 30	57	147	172
	LED	− 2	+ 396	398	+ 50	52	346	108
244	HED	− 31	+ 159	190	+ 20	51	139	149
	LED	− 30	+ 129	159	+ 20	50	109	44
247	HED	− 45	+ 196	241	+ 30	75	166	118
	LED	− 18	+ 300	318	+ 60	78	240	54

The maximum was determined by means of a 3rd degree polynomial fit.

DOY 242 is the brightest TGF with a total fluency (LED + HED) of 280 counts. The light curve morphology is a standard HED TGF light curve with a fast rise time of 57 µs to a peak at 30 µs followed by a slower decay of 147 µs, with a total duration of 204 µs. The LED photons arrive delayed by 25 µs, followed by a rise time of 52 µs to a maximum at 50 µs, and a total duration of 398 µs. While rise times are very similar on LED and HED, the decay times are very different. The HED light curve is essentially symmetric if we exclude 4 photons around 150 µs, while the LED light curve is very asymmetric with a decay duration 7 times longer than the rise time. The longer LED decay times and delayed arrival with respect to the HED photons are a common feature found in our ASIM TGF sample. HED photons (MeV) generated at the TGF ignition layer escape first because of their lower atmosphere scattering cross-section. LED photons generated by the radiation-matter interactions of the primordial MeV photons, arrive delayed consequently with their larger paths in the TGF ignition layer surroundings. This scenario is fully compatible with recent simulations^34^. The 25 µs delay suggests that the first LED photons able to escape came from a region at 8–12 km distance from the RREA (relativistic runaway electron avalanche) TGF core layer with a lateral radius of 2 km^17,35^. The original low energy photons, if any, are absorbed for distances < 8 km.

DOY 244 is the intermediate case with a total fluency (LED + HED) of 193 counts. This TGF has a symmetric light curve with a maximum at 20 µs in both detectors. The TGF duration is very similar in HED and LED with rise times of 51 µs and 50 µs. Both HED and LED observed photons arrive at the same time suggesting that they came for a common envelope surrounding the ignition layer, where Compton, ionization, recombination and pair production thermalize the primordial MeV photons. Beaming is also a possible explanation, see discussion on the Imaging section on beaming effects and source orientation.

DOY 247 is the weakest TGF with 172 counts. Again, LED photons came 30 µs delayed to the HED counts like in DOY 242. HED TGF duration is 241 µs and 318 µs in LED with identical rise times 75 µs and 78 µs respectively.

From this analysis we can conclude that these are three bright TGF of very similar duration and rise times but with a different symmetry in decay times. These differences suggest that a significant fraction of the LED observed photons are generated in layers far away from the ignition RREA core.

### TGF imaging solutions

Before ASIM, no spacecraft observing TGF has been equipped with imaging capabilities. TGF imaging is performed with the Low Energy Detector (LED), which consists of 128 × 128 pixelated Cadmium-Zink-Telluride detectors sensitive in the spectral band from 20 to 350 keV. The capability of locating TGFs is provided by a coded mask as a Multiplexed Perfect Binary Array of 16 × 16 square pixels 46.2 mm × 46.2 mm size, 46% closed (1 mm of tungsten) and 54% open. The mask shadows the detectors at 303 mm distance. Each TGF produces a different spatial pattern of detectors that received photons according to the incidence angle and emission size. This pattern is then deconvolved using the Maximum LIKelihood (MLIK) method^36^ that maximizes the Poisson probability normalized to the uniform distribution. Details about the deconvolution technique are provided in the Methods section.

**Figure 2:**
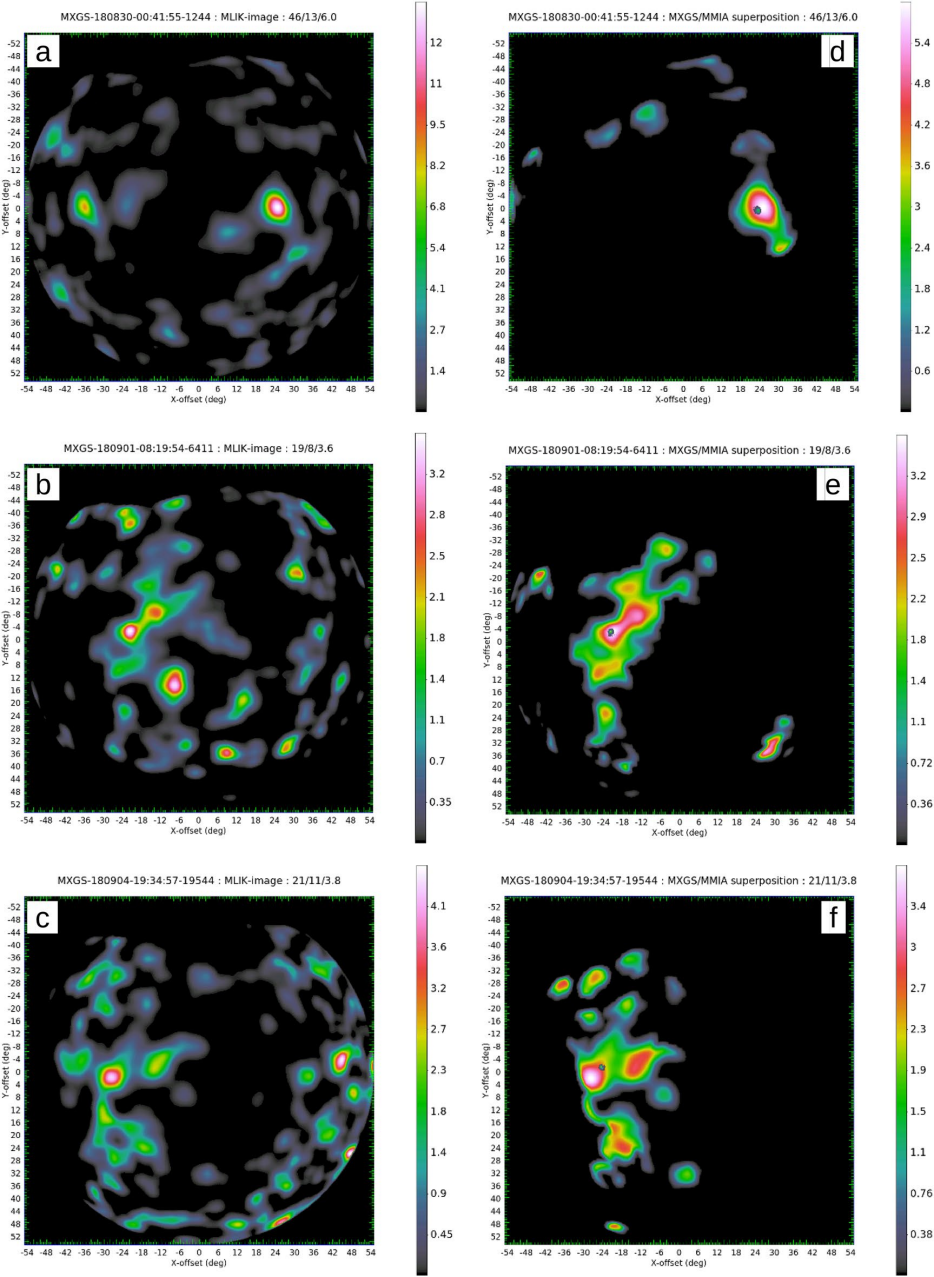
Imaging solution at the MXGS FOV for the three TGF events (**a**) DOY 242, (**b**) DOY 244, and (**c**) DOY 247. The Maximum Likelihood map is in the left column (**a**–**c**) and the signal-to-noise ratio map is shown in the right column (**d**–**f**) where the MMIA optical flash is displayed by a green dot. The ISS forward direction points to the right-hand side.

Figure 2a–c displays the Poisson probability map for the three TGFs. The TGF of DOY 242 has the best imaging solution: larger MLIK value, larger signal to noise ratio (S/N) and smaller peak position uncertainty. It is clear that the solution at the right with a MLIK of 13.4 dominates the map, with a secondary source position at the left with a MLIK of 8.2 (values > 5 are considered good solutions and > 4 acceptable). Taking into account that the MLIK is in logarithmic scale, however, the probability of the secondary source position is 10^5^ times lower. The S/N maps are shown in Fig. 2d–f which includes the MMIA image (green dot) of the associated lightning parent.

TGF DOY 244 (Fig. 2b + e) also has a unique solution but more complex than the TGF DOY 242. The MLIK map has a maximum of 3.5 with a secondary source position (bottom right) with a similar MLIK value. To solve this ambiguity problem, the position of the simultaneous flash located by the MMIA camera is used (green dot in the S/N map). The determination of the source is made using a numerical mask over the solution not compatible with the image of the MMIA camera. After constraining the solution space we get the S/N map of Fig. 2e that determines the imaging solution used for making the projected contours over the geographical map.

The TGF of DOY 247 (Fig. 2c + f) shows a probability maximum of 4.3 in the MLIK map which corresponds to an intermediate imaging quality with a secondary source on the right. The secondary source position lies within the MXGS partially coded field of view (PCFOV, > 40°) and is not compatible with MMIA camera data (as the MMIA FOV is < 40°). The S/N map of the TGF signal confirms the MMIA parent lightning flash location just at the limit of the 1 sigma contour.

## Results: part II: thunderstorms associated with the TGF

### The position of TGF over satellite images and associated lightning detections

**Figure 3:**
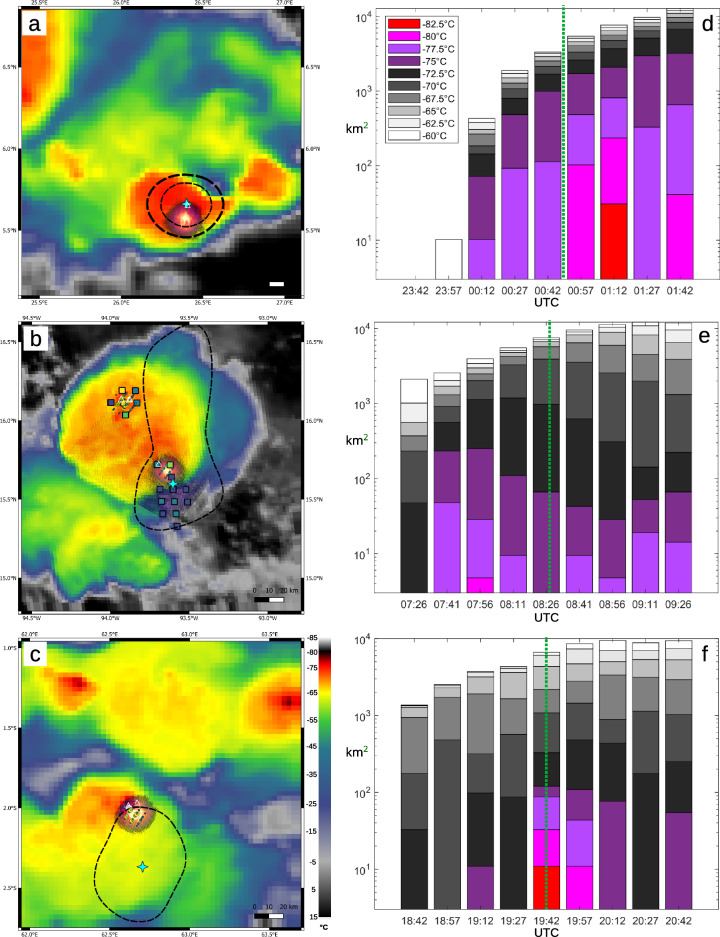
Cloud top temperature with lightning activity detected within 1 s from the TGF (**a**–**c**) and cloud top area evolution (**d**–**f**) for TGF cases DOY 242, 244 and 247 (top to bottom). Overlaid are TGF sigma contours (black lines, interval 1 sigma) and its centroid position (blue star), GLD360 strokes (triangles), ISS-LIS pixels and their intensity (small circles, in (**b**) and (**c**) only), MMIA image pixels (dark purple to yellow shades) and GLM pixels and their intensity (squares, in (**b**) only). In the images, the yellow contour corresponds to − 65 °C, red to − 70 °C, and black to − 80 °C. Both ISS-LIS and GLM have been converted to energy density at the cloud top, following^38^, and the color scale spans the range 0–100 J km^−1^ (in 2 ms exposures). The MMIA color scale spans 0–1000 J km^−1^ (accumulated during its 83 ms exposure).

The TGF S/N maps have been converted to TGF footprint projections over the cloud top temperature images by Meteosat and GOES satellites (dashed black contours in Fig. 3a–c).We overlaid the associated lightning locations provided by various systems, described in more detail in the Methods section. It includes the 337 nm wavelength parent lightning flash image by ASIM (MMIA), the flash pixels of the Lightning Imaging Sensor on the ISS (LIS, small circles, for DOY 244 and 247), the Geostationary Lightning Mapper (GLM, colored squares, for DOY 244 only) and the GLD360 global lightning detection network (white triangles) all within 1 s of the TGF time. So, not all depicted lightning processes were active at the time of the TGF itself. The area size (magnification) is kept the same for all three events, approximately 190 by 190 km. The Meteosat image, MMIA image and TGF footprint have been corrected for the horizontal shift arising from the angle under which the cloud top is projected on the ground when seen from the satellite, known as parallax error (typically 5–8 km). The ISS-LIS and GLM data already include a parallax correction. Furthermore, the TGF footprint and MMIA image have been corrected for meridional angle drift of the ISS along its orbit due to the Earth’s rotation^37^. After these corrections, the parent flashes of the three TGFs mapped by MMIA, as well as ISS-LIS, appear collocated with the coldest (overshooting) cloud tops of convective cells. The GLD360 detections in all three cases are offset slightly to the north. Supplementary Fig. S1 shows the location of the TGFs relative to World Wide Lightning Location Network (WWLLN) cloud-to-ground lightning locations 15 min before and after the event, allowing to assess the overall activity level near the event location.

The bottom section of Table 3 summarizes the TGF longitude, latitude, position error radius, distance between TGF and the ISS and TGF distance to the ISS footprint as projections of the imaging solutions at the MXGS FOV.

**Table 3:** Imaging results.

DOY	242	244	247
Counts used for imaging	59	27	32
Source counts	46	19	21
Energy (keV)	45–163	50–183	54–165
MXGS field of view			
MXGS FOV X-offset (degrees)	+ 24.727	− 21,603	− 27,615
MXGS FOV Y-offset (degrees)	+ 00.114	− 02.222	+ 02.157
Source angular distance (degrees)	25	22	28
Maximum Likelihood (MLIK)	13.4	3.5	4.3
S/N ratio at 1 sigma	6.0	3.7	3.7
Source location error radius (degrees)	1.9	5.7	4.0
MXGS footprint solutions			
TGF longitude (degrees)	+ 26.400	− 93.603	+ 62.706
TGF latitude (degrees)	+ 5.661	+ 15.599	− 02.368
TGF position error radius (km)	14	43	32
TGF distance to the ISS (km)	425	431	458
TGF distance to ISS footprint (km)	161	176	227

The event of DOY 242 over Central Africa (Fig. 3a), with the best imaging (1.9° Source Location Error Radius, Table 3), has the smallest TGF Position Error Radius (PER) of 14 km at 1 sigma. The MMIA image is situated over the highest cloud tops of the cell, while the TGF 1-sigma contour line is centered over the width of the cell and includes the associated flash location. No WWLLN or GLD 360 sferics were found in association with the TGF within +− 30 ms around the TGF time although there is one GLD360 stroke detected within +− 1 s. ISS-LIS did not reproduce this flash.

**Figure 4:**
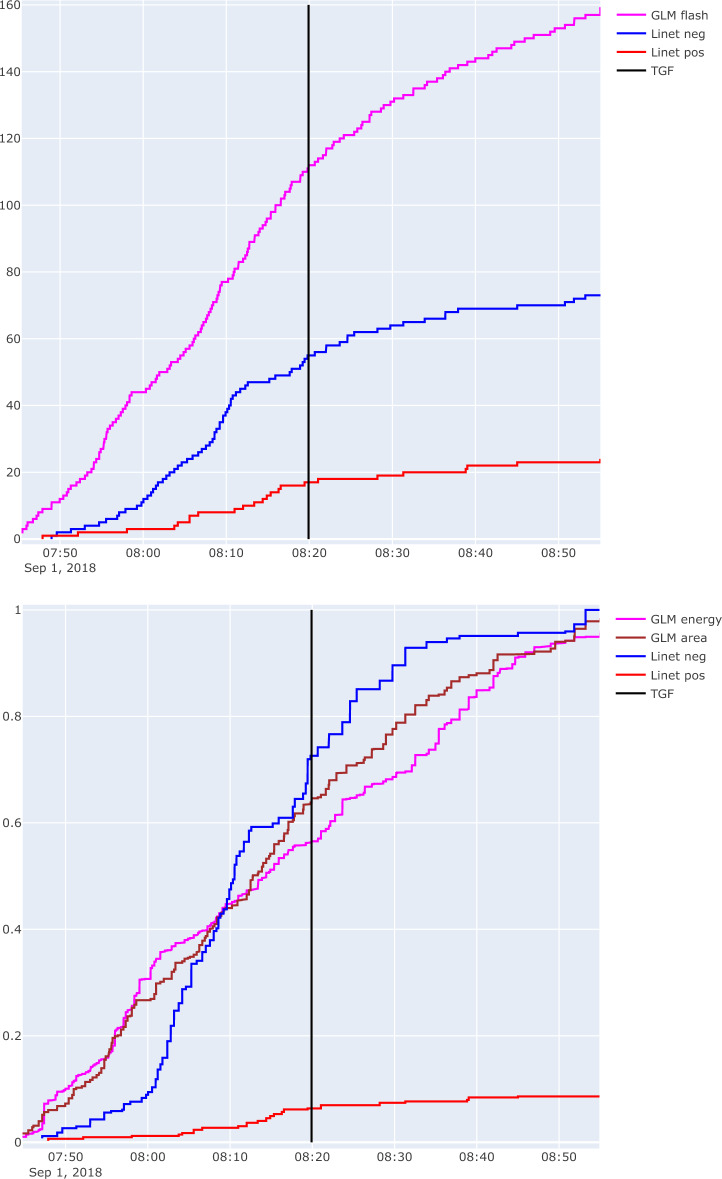
Cumulative flash rate evolution for the DOY 244 event over Mexico. (**a**) Cumulative flash counts of GLM and LINET by polarity. (**b**) Cumulative energy and area for GLM groups and accumulated peak current of LINET negative and positive strokes, normalized by their maximum values (except positive, normalized by the negative count to maintain the relative proportion).

The 1-sigma contour of DOY 247 over the Indian Ocean (Fig. 4c) is wider (PER 32 km) and appears over anvil cloud offset to the south of the lightning activity in DOY 247, which is situated at the highest cloud top. However, the sigma contour does include the possibility of the TGF being emitted by the convective cell.

The 1-sigma contour of DOY 244 at the Pacific coast of Mexico (Fig. 4b) with a PER of 43 km spans across a large thunderstorm system with multiple active convective cells. A large lightning cluster of 70 by 100 km is indicated by the WWLLN sferics (Fig. S1b). During the +−1 s displayed, two flashes were active in this system. The flash that corresponds in time with the TGF occurred in a cell at the southern edge of the large expanding anvil, as detected by MMIA, ISS-LIS, GLD360 and GLM. The other flash was not covered by the 1-sigma contour. This cell reached its highest tops in the GOES image 15 min before the TGF event. At the time of the TGF the overshooting tops form a cold ring around a 4 K warmer spot which was at the location of the initial overshooting top. For this figure a MMIA image was selected closest to the time of the TGF, however, MMIA also has a frame that detected the lightning flash collocated with the GLD360, ISS-LIS and GLM detections in the cell in the northern sector of the anvil cloud. Both GLM and MMIA show low luminosity pixels over the area just south of the anvil cloud (it is recommended to zoom in). These indicate a reflection of light coming from the side of the updraft tower, via the surrounding lower clouds or sea surface.

The 1 sigma uncertainty line of this TGF extends up to 100 km to the north of the TGF and MMIA positions, towards the WWLLN lightning cluster center, suggesting this TGF is a very large extended source, with a relevant fraction of the LED observed counts coming from layers far away from the TGF ignition layer (tens of km).

### Satellite cloud top evolution

Animating the satellite images reveals in all three cases a convective cell that develops 45 min to less than 15 min before the TGF, with cloud tops becoming colder at the location of the associated lightning event, and expanding. This can be visualized by a cloud top temperature area evolution, shown in Fig. 3d–f. These are stacked histograms of area by temperature interval as in Ref.^41^, made by creating a region of interest around the relevant thunderstorm cell and anvil cloud at each time step. Here, the growth is clearly visible in the DOY 242 (d) and 247 (f) cases, with the cell continuing to intensify dramatically after the TGF time in the case of DOY 242. The new cell growth in the DOY 244 case (e) is embedded within the southeast section of the cloud anvil. A strong overshooting top appeared 15 min before the TGF (at 15.79°N, 93.7°W) and started to collapse, which then produced the TGF. This cell does not show up in the evolution graph because at the same moment a larger convective region with high cloud tops in the northwest was decaying. These developments could not be separated during the selection of the region of interest.

### Lightning flash rate evolution

The cases of DOY 242 and 247 occurred over central Africa and the Indian Ocean, where detection efficiency of available global lightning detection networks like WWLLN is limited to the strongest strokes because ELF/VLF detection sensors are widely spaced. The majority of lightning activity in storms is missed, as are details of the flash rate evolution. However, the case of DOY 244 occurred at the southwest coast of Mexico, within the range of GLM and a regional LINET network with a very high flash detection efficiency. This allows a quantitative study of the flash rate. Here, we selected a region of interest that isolates best the relevant lightning activity associated with the convection that triggered the TGF (the area within the rectangle 93.75–93.3°W, 15.55–15.78°N). The method of cumulative flash counts^42–45^), a “stair step” graph, has the advantage of showing the native cadence of flash occurrence relative to the time of the event, with a time resolution better than the 1–5 min of traditional flash rate bar graphs. Figure 4a shows the cumulative flash counts of GLM and LINET flashes. For this, the native GLM flashes are used, while LINET flashes have been created only of strokes that were not preceded by another stroke within 0.5 s in the region of interest. Negative and positive polarity have been considered separately and no association has been made between GLM and LINET events. Over the period 07:45–09:00 UTC the flash counts of GLM reached slightly over twice those of LINET, as it detected more intracloud flashes. A few things can be noted: The TGF occurred when GLM rates were changing from a higher to a lower mean rate (3.7 per minute to 1.1 per minute in this region). The LINET negative flash rates were less steady with a notable increase around 08:10 UTC, but then going almost silent for the next few minutes (08:13 to 08:18). Positive rates, likely intracloud flashes, remained active during these minutes, but ceased once the negative rates picked up again.

This evolution is magnified in Fig. 4b, which shows GLM accumulative group energy and area, and LINET accumulative stroke peak current, normalized by their final value at 9 UTC. A GLM group is a 2 ms image as part of a flash, often spaced by tens of milliseconds (as shown for example by Ref.^38^), much like LINET strokes make up a flash. Here, the GLM group energy appears to weaken in the minutes before the TGF, and then pick up again. The flash that started simultaneously with the TGF is not remarkable by itself (small step in area and energy). The LINET negative peak current evolution is characterized by a sudden lull from 08:13 to 08:18 (5 min) and a sharp increase in the minute before the TGF occurred. GLM energy per group (and flash) became temporarily very small in the 2 min before the TGF simultaneous with the restarted LINET negative rates.

The LINET cloud-to-ground flash 31 s before the TGF was the largest event during this period, consisting of 8 strokes, the first one being − 93 kA. This flash accounted for 5% of the accumulative stroke peak current (1/20th part of the energy as 1 of 74 flashes).

In summary, there seems to be a possible development in the electrical configuration of the storm where negative flash rates ceased for 5–6 min, while intracloud flashes continued and became more detectable as positive flashes, a period that ended with the production of the strongest negative cloud-to-ground flash and the TGF, followed by an overall reduction of flash rates but optically strong GLM flashes. It must be noted that a − 93 kA cloud-to-ground stroke and a multiplicity of 8 strokes in itself is not a rare event, but it appears to be evidence of a strong build-up of negative potential in the cloud, which may be needed for the TGF as well.

### Meteorological environment of the storm

Vertical profiles of atmospheric variables have been retrieved for each case from the ERA5 reanalysis data^39^. In the DOY 242 case there is no location with very small CIN, although there is CAPE. It is possible that the environment was affected by previous convection. For each profile, various parameters have been calculated as in our statistical study related to gigantic jets in Colombia^40^. A selection of those is presented in Table 4. Like TGFs, gigantic jets are electrical discharges with energy transported to the higher atmosphere, and are mostly confined to tropical meteorological environments. Although gigantic jets may require different electrical conditions for their initiation than TGFs, provided by a specific thunderstorm environment, the study identified very precisely the range of parameters within the broader tropical environment that may affect the thunderstorm processes enough to enable these very rare discharges. The parameters which had significantly different values in profiles associated with gigantic jets compared to a set of null cases were mainly those associated with microphysical processes, such as Warm Cloud Depth (WCD): the vertical distance between − 10 °C isotherm altitude and the Lifting Condensation Level (cloud base). For a detailed explanation of the parameters we refer the reader to that work. Given that we only have three example events and no null cases in this study, we can only compare the values of the parameters of each case against the baseline provided in Ref.^40^.

**Table 4:** Selection of parameters (described in *van der Velde *et al*.*^40^ for gigantic jets) derived from ERA5 proximity profiles and their values in the three TGF cases.

Parameter	DOY 242	DOY 244	DOY 247
	0000 UTC	0700 UTC	1700 UTC
CAPE (J kg^−1^)	**1613**	**1563**	**766**
CIN_ML_ (J kg^−1^)	*− 62*	** − 10**	** − 8**
‍MPL-EL (km)	**3.32**	3.53	**2.41**
DD Δ*θ*ES _950 hPa_ (K)	27.0	**21.1**	**13.8**
RH _925 hPa_ (%)	82.4	75.1	**94.6**
Z_-10 °C_ (m)‍	6500	6490	6656
WCD_-10 °C_ (m)	5618	5828	**6136**
DZ_-10–50 °C_ (m)	**5363**	5510	**4985**
WCDRAT (ratio)	1.05	1.06	**1.23**
CON_-10 °C_ (g kg^−1^)	11.7	12.3	12.3
CON‍_-10–30 °C_ (g kg^−1^)	3.8	3.9	**3.0**
WCCRAT (ratio)	3.04	3.14	**4.08**
‍WCCRAT*EL	42.9	43.5	**52.8**
‍MSH _1000–600 hPa_ (10^−3^ s^−1^)	**3.4**	**3.3**	**2.5**
SH _150–100 hPa_ (m s^−1^)	*19.1*	*1.8*	*16.9*

When the value is closer to that of a gigantic jet environment, it is printed bold. When a value is neither close to the mean of a gigantic jet nor a null case environment, it is printed cursive.

In short, the values of DOY 247 correspond with a typical gigantic jet environment in all aspects (weaker instability, stronger warm cloud parameters, weaker vertical wind shear), while those of DOY 242 and 244 mostly do not. It can be noted that the cloud top level wind shear (100–150 hPa) could be called quite strong in two of the TGF cases (17–19 m s^−1^), but given the typical variance of that parameter in the gigantic jet study these are not unusual. Although there are only few data points, these do not point at one particular environment or single parameter with elevated values in all three cases.

## Discussion

The TGF locating capabilities of the ASIM MXGS have been demonstrated for three bright TGF detected by ASIM’s High Energy Detector (HED), and imaged by the Low Energy Detector (LED) with its coded mask. Taking benefit of the precise TGF positions, the meteorological characteristics associated with these events were discussed. The three TGFs have very similar incidence angles and source-observer distances to the MXGS allowing us to perform comparative studies of the TGF properties.

The imaging analysis showed that the source location error radius at 1 sigma at the MXGS fully coded FOV for the DOY 242 solution is 1.9° which is the best of all imaging MXGS solutions. This value is in good agreement with the theoretical MXGS angular resolution of 1.3° for a bright TGF with 100 imaging counts^32^. In the case of DOY 242 we have 59 imaging counts. It can be considered the operational MXGS imaging resolution for bright TGF at the FOV center (25°). For the DOY 247 with 32 imaging counts (a typical medium quality TGF) and DOY 244 with 27 imaging counts, the resolution is degraded to 4.0° and 5.7° respectively at the MXGS FOV. The larger apparent source size in TGF 247 is consistent with the reduced number of imaging counts available. The Source Location Error Radius (SLER) is larger in the TGF 244 because of both the reduced number of counts and the extended, asymmetric source size clearly seen in the footprint map.

The HED and LED showed similar rise times, on average 60 ± 16 µs and 61 ± 12 µs respectively, but LED decay times lasted 1 to 7 times longer than HED. The larger difference suggests that for the asymmetric TGF, a significant fraction of the LED observed photons are generated in layers far away from the ignition RREA core. LED counts also arrive 25 µs later indicating that they are generated in an extended region compared to the HED ones, at distances of 8–12 km. These values will allow us to define better the origin conditions and characteristics of the TGF source^46^.

Although the imaging positioning uncertainties are larger than lightning position determination by sferics or cameras, which are typically less than a few km, the TGF and lightning parent are now determined independently in space and time without any assumption. In past studies, the position of a TGF and its parent thunderstorm was impossible to determine if it could not be matched to within milliseconds of a detected lightning stroke reported by ground-based lightning detection networks.

We used the example TGFs to investigate the parent thunderstorm and lightning activity. In all 3 cases, the TGF was produced by a thunderstorm cell with cloud tops overshooting their surrounding anvil cloud at the time of the event, but the timing to the most intense state of convection, in terms of cloud top height and its area, varies. This is a result that also has been reported by other studies^22,25^. Given that only one TGF is typically detected during the 3 min that ASIM observes the storm, but more TGFs may have been produced during its life cycle, the state of the storm at the exact moment of TGF detection may not be the unique condition in which all TGFs are necessarily produced. The analysis of lightning activity has been a subject of several studies, with the general conclusion that TGFs tend to occur most often around the peak in cloud-to-ground flash rates^25^ or later^26,27^). We analyzed one of the cases for which GLM and a regional LINET lightning detection network data were available, with a higher detection efficiency than worldwide networks as WWLLN. This event confirms a timing when GLM flash rates in this section of the thunderstorm complex were starting to decrease and their energy increase. Before the GLM per-flash energy increased, there was a period of a few minutes preceding the TGF when the GLM optical energy per flash was very low. LINET negative cloud-to-ground flashes suddenly stopped being produced 7.5 min before the TGF, which seems in line with extended charging time reported before^28^, while some positive ones (perhaps intense intracloud flashes) were detected during that time. The TGF event occurred when the negative flashes picked up again since 1–2 min, and was triggered 31 s after an 8-stroke − 93 kA flash.

Some previous studies have mentioned that the meteorological conditions and resulting convection were not particularly exceptional (e.g. Refs.^22–24^). We confirm a clear relation to recent convective cell development in the three cases of our study, like is typical also for gigantic jets which share the overall tropical distribution with TGFs. A higher TGF production efficiency per lightning flash was found in maritime tropical environments^29^. Similarly it was noted^40^ that warm maritime tropical profiles (very humid and cooler low levels with warmer mid levels) are associated with gigantic jets. Like gigantic jets, TGFs appear to occur before or during negative-upward leaders^12–14^ and would need a significant upward directed electric field^8^, so it appears that similar meteorological circumstances may benefit both phenomena, even if the details of the parent discharges differ. However, here we found no unanimous endorsement of either maritime, humid type of environment or the drier, more unstable type of profile of null cases^40^. In two of these cases there was quite strong vertical wind shear at the cloud top level. Note that studies of the TGF production environment are complicated by lack of permanent observation to obtain a set of null cases for reference. Cases that include one TGF by no means confirm that such storms were more prolific in producing TGFs than other storms, considering the luck factor involved in catching that TGF during the satellite overpass.

Following the path opened by this paper, future studies can be performed using the global database of additional ASIM TGFs with a total number of 73 additional ASIM TGFs covering a wide range of angular distances up to 54° off axis (source to observer distance up to 700 km). The ASIM mission will be operational until late 2025. This growing database enables statistical studies of the parent thunderstorm properties, using in the near future also the Meteosat Third Generation and its Lightning Imager.

## Methods

### Lightning data

Lightning data from the Vaisala Global Lightning Dataset (GLD360)^47^, the optical Lightning Imaging Sensor on the International Space Station (ISS-LIS)^48^ and the Geostationary Lightning Mapper (GLM)^49^ onboard the GOES-16 have been used to associate each studied TGF event with lightning flashes. For the DOY-244 case, a flash rate evolution has been analyzed in detail using data from GLM and a regional LINET network^50^ in Mexico. Data from the World Wide Lightning Location Network (WWLLN)^51^ is used for initial verification of TGF and MMIA positions. The GLD360 network, after its 2015 upgrade, is reported to have 75–85% detection efficiency compared with a large national lightning detection network^52^. A mean location accuracy improvement to 1.5–2 km was announced by Vaisala in 2018. WWLLN is a result of academic cooperation, operating in the 3–30 kHz range, detecting mainly strong strokes with a typical precision of 3–30 km^53,54^ with low general detection efficiency in remote areas, e.g. 6% in central Africa^55^. A regional LINET VLF/LF network with station baselines < 250 m is capable of locating lightning with a typical location error of 150 m and a detection efficiency over 90%. It has the capability to detect intracloud pulses and their height^50^. Of the satellite-based detectors, ISS-LIS has a pixel footprint of about 4.5 km at nadir, while GLM has a footprint of 8 km at nadir. Both detect luminosity changes in the 777.4 nm oxygen triplet band at 2 ms temporal resolution. The detection efficiency of GLM, on average over 70%, varies between 20 and 95% depending on the rate and size of flashes^56,57^.

### Cloud top temperature and meteorological data

For the analysis of the cloud tops of the thunderstorms associated with the TGFs we use data from meteorological geostationary satellites. For DOY 247, we have used data of the channel 9 (10.8 μm) of the Meteosat-8 (Indian Ocean, 41.5°E). In the case of DOY 242, satellite data from the channel 9 (10.8 μm) of the Meteosat-11 has been employed (Africa, 0°E) and for DOY 244, the GOES-16 band 13 (10.3 μm) has been used (Americas, 75.2°W). Furthermore, we use meteorological data from the ERA5 reanalysis (Copernicus Climate Change Service (C3S)^39^: ERA5: Fifth generation of ECMWF atmospheric reanalysis of the global climate available at pressure levels and hourly intervals at a spatial grid of 0.25° to analyze the vertical temperature, humidity and wind profiles for each case. Using the same methodology and meteorological parameters as in Ref.^40^ for the statistical study of gigantic jet environments, we selected one proximity profile close to the time of the TGF-producing storm, at a location within 0.5 degree latitude/longitude with the smallest amount of convective inhibition. That study also serves as a framework for the interpretation of values of convective parameters for TGF production in the present study.

### ASIM data and instrumental setup

The ASIM payload has two instruments: MXGS^31^ and MMIA^32^. The MXGS is an instrument designed for TGF, detection, location and spectral analysis. It consists of the Low Energy Detector (LED) and a High Energy Detector (HED). LED is an evolution of the technology used in the *Minisat 01*^58^ and *INTEGRAL*^59^ space missions for gamma ray astronomy, with a very wide operational field of view (− 40° to + 40° fully coded plus an extra 15° partially coded). It consists of 128 × 128 pixelated Cadmium-Zink-Telluride (CZT) detectors sensitive in the spectral band from 20 to 350 keV, while HED consists of 12 Bismuth-Germanium-Oxide (BGO) detector bars each coupled to a photomultiplier tube sensitive from 300 keV to 30 MeV. The capability of locating TGFs is provided by a coded mask as a Multiplexed Perfect Binary Array of 16 × 16 square pixels 46.2 mm × 46.2 mm size, 46% closed (1 mm of tungsten) and 54% open. The mask shadows the LED detector at 303 mm distance.

MMIA consists of two cameras continuously recording 12 frames per second in the 777.4 nm and 337.0 nm bands (4–5 nm wide) together with 100 kHz photometers for the same two bands and an additional photometer in the 180–230 nm band. The camera pixel footprint at the ground in the nadir is approximately 400 m.

The three TGFs presented in this work were detected by the HED instrument using the 0.3 ms window at a very early phase of ASIM operation in August and September 2018. Once the trigger was declared, 2 s of data were recorded, one before and one after the trigger time (TT). MXGS triggered also MMIA photometers and cameras. The full set of data are stored at the ASIM Science Data Center (ASDC) as a Level 0 products. Table 1 lists the TGF ID, trigger time, duration and ISS position. The “OBS” number is the internal ASDC ID useful for data acquisition at the ASIM database. Once the TGF was declared by ASDC, the University of Valencia (UV) software analyzed the raw LED and HED data to identify hot pixels, multi hit events and BGO overflow counts. The operational LED energy range is 45 keV to 350 keV. The lower limit is defined by the operational CZT threshold and the upper limit comes from the high-count rates generated by the multi hit events and partial deposits in the CZT crystals from high-energy incoming photons (MeV).

The imaging deconvolution is performed by using the Maximum Likelihood method that maximizes the Poisson Probability^36^. MLIK is the computed Maximum LIKelihood Poisson probability for any position at the detector layer normalized to the Poisson probability for a uniform distribution in logarithmic scale defined as:

$${\text{MLIK}}\left( {{\text{x}},{\text{y}}} \right) \, = {\text{ log }}\left( {{\text{ P}}\left( {{\text{S}}\left( {{\text{x}},{\text{y}}} \right) > 0} \right){\text{/P}}\left( {{\text{S}}\left( {{\text{x}},{\text{y}}} \right) = 0} \right)} \right),$$

$${\text{with}}\, {\text{C}}\left( {{\text{x}},{\text{y}}} \right)\, = \,{\text{M}}\left( {{\text{i}},{\text{j}}} \right)\,*\,{\text{S}}\left( {{\text{x}},{\text{y}}} \right)\, + \,{\text{B}}\,.$$

S(x,y) are the source counts before the mask for a given position (x,y), M(i,j) is the mask pattern, B is the imaging background and C(x,y) are the source counts detected. When S = 0, C(x,y) = B is constant and consequently the Poisson probability is the same for all the possible (x,y) positions in the MXGS Field of View (FOV). S = 0 is equivalent to no imaging solution. MLIK values larger than 5 indicate good imaging solutions, between 4–5 acceptable imaging solutions, and below 4 low quality imaging solutions. Table 3 shows for each TGF the number of counts used in imaging and the source counts accepted. The difference are imaging background counts. Also listed are the energy range used, the TGF x and y axis position and source angular distance at the MXGS Field Of View (FOV). The last three rows contain the computed MLIK value, Signal to Noise Ratio (S/N) and the Source Location Error Radius (SLER) at 1 sigma.

TGF imaging is not a trivial task. In addition to the intrinsic uncertainties associated with the imaging technique, there are two additional sources of uncertainty: the high TGF peak fluxes and the source size/altitude.

In the first 2 years of ASIM operations and for the 117 TGFs with imaging solutions, HED peak fluxes vary from a minimum value of 0.2 to a maximum of 3.4 counts µs^−1^. 55 TGFs have fluxes > 1 count µs^−1^ and 62 TGFs have fluxes below that limit. For the bright TGFs, the partial deposits of the incoming MeV photons increase the number of counts recorded in the CZT crystals. One can easily identify in the raw LED spectrum the concentration of multi hits and partial deposits at energies above 350 keV, but they are also present at the imaging energy range (50–200 keV). The prescription to solve the problem is to use anti-coincidence techniques LED/HED to clean the imaging counts. Since 2020, an anti-coincidence system is incorporated in the Mathematical Data Analysis Pipeline (MDAP) imaging software. For the 3 TGFs in this work, with peak LED fluxes of 2.4, 2.0 and 1.5 count µs^−1^ anti-coincidence LED/HED was used.

During early phases of ASIM development, the question of the source size was addressed to optimize the ASIM imaging performances. Assuming the RREA mechanism for TGF generation^9,16^, the avalanche development happens in a reduced cylindrical volume with a characteristic length L < 200 m and a lateral radius R >  > L. For a TGF observed by BATSE at 15 km altitude^25^ a lateral radius of 1 km was estimated. Photons with energies < 1 MeV are generated by the interaction of the primordial MeV photons with the atmospheric components via Compton scattering, ionization, recombination and pair production processes. So the LED imaging photons with energies from 50 to 200 keV used in imaging are generated in a larger volume surrounding the TGF ignition layer. The smallest TGF apparent source size as resolved by LED is estimated to be 1.3° in the MXGS FOV for a bright TGF at 15 km altitude^31^. The observed source size will increase for deeper TGF and/or tilted beam geometries with respect to the observer.

The last element to take into account is the modulation capability of the 1 mm thickness tungsten (wolfram) mask. Photon absorption is larger than 98% up to 120 keV dropping to 80% at 200 keV and < 40% at 350 keV. The use of LED photons above 200 keV for imaging purposes is not recommended, because these non-modulated photons increase the imaging noise and consequently S/N is degraded.

Since the first ASIM TGF detection in 2018, the imaging solution was improved many times according with the development of the MDAP imaging software. We use the most recent September 2022 version.

### Supplementary Information

Supplementary Figure S1.

## Data Availability

TGF and MMIA data and GLD360 data associated with events are available from the ASIM Science Data Centerhttps://asdc.space.dtu.dk. ISS-LIS data is available online from the NASA Global Hydrometeorology Resource Center DAAC, Huntsville, Alabama, U.S.A. 10.5067/LIS/ISSLIS/DATA109. We obtained NASA GOES and GLM data from the GOES-16/17/18 download service on Amazon by the Department of Atmospheric Science of the University of Utah. Meteosat data is available from the EUMETSAT Data Store. ERA5 data is available from the European Union Copernicus Climate Data Store. LINET data can be requested at Nowcast GmbH.
