# Supplementary material for: Imaging of 3 bright terrestrial gamma-ray flashes by the atmosphere-space interactions monitor and their parent thunderstorms
Source: Sci Rep. 2024 Mar 23;14:6946. doi: 10.1038/s41598-024-57229-1 (PMC10960811; doi:10.1038/s41598-024-57229-1)
Supplement: Supplementary file 1 — Supplementary Figure S1. [file 41598_2024_57229_MOESM1_ESM.pdf]

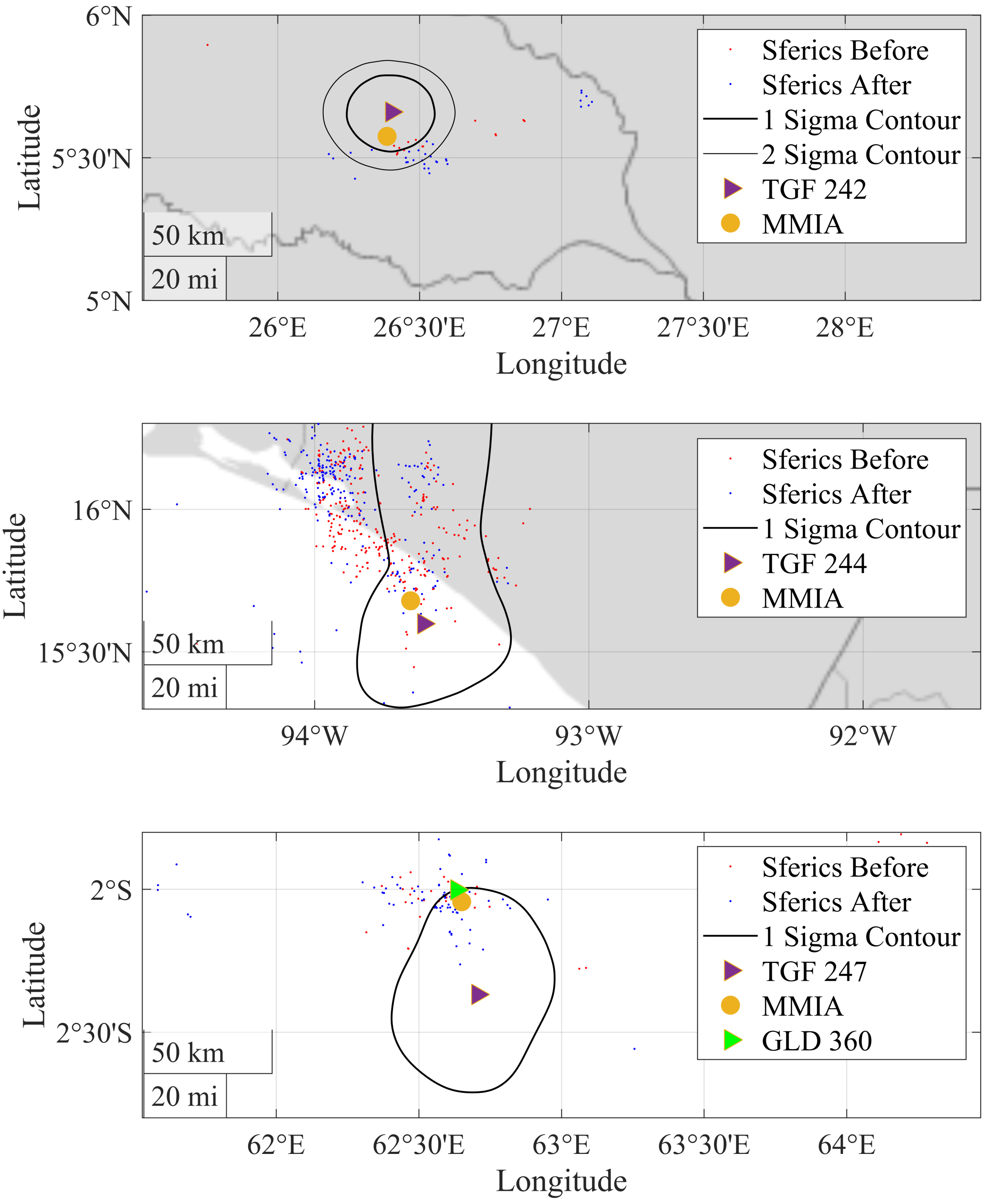


**Figure S1.** TGF event footprints, maxima (purple triangles), and MMIA centroids relative to WWLLN lightning detections during the 15 minutes before (red) and after (blue) the event.
